# Behaviors and Attitudes of Obstetricians in Turkey Related to Cord Clamping, Cord Milking, and Skin-To-Skin Contact

**DOI:** 10.7759/cureus.16227

**Published:** 2021-07-07

**Authors:** Demet Aydogan Kirmizi, Emre Başer, Melike Demir Çaltekin, Taylan Onat, Mustafa Kara, Ethem S Yalvac

**Affiliations:** 1 Obstetrics and Gynecology, Yozgat Bozok University, Yozgat, TUR; 2 Obstetrics and Gynecology, Kırşehir Ahi Evran University, Yozgat, TUR

**Keywords:** birth, cord clamping, cord milking, obstetrician, skin-to-skin contact

## Abstract

Objective

This study was planned to evaluate obstetricians' practices of umbilical cord clamping, milking, and skin-to-skin contact applications and to determine the related variables.

Material and methods

A total of 522 obstetricians participated in the study. Participants were reached via the internet and a 15-item questionnaire was applied about umbilical cord clamping, cord milking, and skin-to-skin contact. Mann-Whitney U test and Student's t-test were used for continuous data and Chi-square test or Fisher's exact test for categorical data in determining the difference between groups. For the multivariate analysis, the possible factors identified with univariate analyses were entered into the logistic regression analysis to determine further independent predictors of delayed clamping. Statistical analysis was performed using the SPSS software (version 20, IBM Corp, Armonk, USA).

Results

It was determined that 234 (44.8%) of the participants clamped the umbilical cord early whereas 288 (55.2%) clamped it late. It was found that the delayed clamping rates of physicians working in public hospitals were significantly lower than those working in private (p<0.001). It was observed that 132 of the participants (25.3%) did not milk the cord and 180 (34.5%) of them applied it continuously, and no significant difference was found between physicians working in the public and private sectors (p=0.130). It was observed that 384 (73.6%) physicians applied skin-to-skin contact. In the multivariate regression analysis, it was determined that working status in a private hospital with a 3.6 odds ratio (OR) (95% CI = 2.0-6.3) and a low number of patients examined daily with a 1.2 OR (95%CI= 1.0-1.4) were the most important independent factors affecting the choice of delayed clamping.

Conclusion

It is seen that the most important parameter affecting the birth practices of physicians in our country is the employment status in public or private institutions. The age and professional experience of the physicians who clamp the umbilical cord late were found to be higher. Obstetricians are at the key point in obstetrics practice, and the experience of physicians and the type of institution they work with affect these practices.

## Introduction

"When should we clamp the cord after birth?" has been a question that has been asked since the time of Aristotle [1]. Studies conducted from the 1950s to the early 2000s reported that approximately 100 ml of blood was transferred from the placenta to the newborn in the first 3 minutes after birth and 90% of this transfer occurred in the first few breaths in term babies [2-4]. Due to these observations and the lack of specific recommendations regarding optimal timing, early clamping of the umbilical cord was applied.

In the standard practice of those years, early clamping was defined as within 1 minute, and late clamping as 5 minutes. In the 21st century, it is seen that the results obtained by conducting more randomized controlled studies on blood volume, oxygenation, arterial, venous pressure, and blood flow in term and preterm babies have created a trend towards delayed clamping [5, 6]. Current recommendations are to delay clamping by >30 seconds [7], 30-60 seconds [8], at least 60 seconds [9], or 30-180 seconds [10]. Nowadays, it is stated that delayed cord clamping increases hemoglobin levels in term fetuses and improves iron stores in the first few months of life, decreases the rates of intraventricular hemorrhage and necrotizing enterocolitis in preterm fetuses, and causes less need for blood transfusion. However, there seems to be hesitation about the application of delayed clamping as resuscitation might also be delayed in preterm fetuses, and the concerns about polycythemia and increased jaundice due to excessive placental transfusion arise.

Some studies have indicated that when emergency neonatal resuscitation is required or when maternal hemodynamic instability is observed, umbilical cord milking or stripping can be applied instead of delayed clamping. However, there is no consensus on this issue either [11, 12]. In the guidelines created by neonatologists, the International Liaison Committee on Resuscitation 2015 (ILCOR) recommends clamping the cord after 30 seconds at the earliest in all term and preterm babies that do not require resuscitation, but states that there are doubts about the method of stripping/patting in premature babies who need resuscitation [7]. As a result, today, organizations such as the American Congress of Obstetricians and Gynecologists (ACOG), World Health Organization (WHO), and ILCOR recommend delayed clamping of the cord, but state that there is insufficient evidence to support or reject umbilical cord milking in term or premature babies [7, 13, 14].

It is a well-known fact that birth practice has changed nowadays. In this physiological process, practices such as mother and father's participation in birth, doulas (trained lay birth assistants), and skin-to-skin contact are directly accepted by some experts, and for others, they stand out as approaches that are avoided by the thought that they carry fetal and maternal risks. But the only real known fact is that we obstetricians are at the center of birth practices.

Despite various recommendations and regulations, it is observed that there are differences in obstetricians' birth practices and cord clamping times all over the world. It has been shown in some studies that these differences are affected by various factors such as health management systems and physicians. However, in our country, Turkey, there is no study evaluating the factors affecting these attitudes and behaviors. In our country, there is no protocol suggested by the Ministry of Health or any other organization about cord clamping time. Obstetricians, apply clamping and milking methods according to their own experience. The purpose of this study is to evaluate behaviors and attitudes of obstetrics and gynecology experts in Turkey related to the applications of cord clamping time, cord milking, and skin-to-skin contact.

## Materials and methods

This cross-sectional study was conducted in the Yozgat Bozok University Faculty of Medicine. The data were obtained from obstetricians via a questionnaire-based survey that was carried out between May 2020 and September 2020 about umbilical cord clamping, cord milking, and skin-to-skin contact after birth. The study had been reviewed by the appropriate ethics committee and had been performed in accordance with the ethical standards described in an appropriate version of the 1975 Declaration of Helsinki, as revised in 2000. Ethical approval was obtained for this study (2017-KAEK-189_2020.03.09_09).

Sampling effects were calculated to weigh for the relationship between umbilical cord clamping, milking, and skin-to-skin contact characteristics with the institution where specialists work. According to official statistics offered by the Turkish Medical-Health Sciences Education Council, a population of 6,163 obstetricians (specialists) was registered in Turkey. To estimate the sample size, the following criteria were considered. As it was a multiple-choice questionnaire in which the prevalence of each response option was unknown, a prevalence of 50% was used for being the criterion that requires the largest sample size. Additionally, a confidence level of 97% was stated and precision or absolute error of 5%, giving a minimum sample size of 438 study subjects. In anticipation of a 20% non-response rate and 20% missing values, the minimum sample size increased to 730 participants. On the basis of this estimate, we decided to enroll 770 participants, to account for a 5% loss of information due to contingencies. The participants were reached via the internet. Obstetricians who did not agree to participate in the study and did not actively work in the field (who did not deliver) were not included in the study.

Informed consent of the obstetricians was first obtained for the study. If they agreed to participate, a 15-item questionnaire prepared by us based on previously published studies on the same subject was applied[15, 16].

Participants' demographic information such as age, gender, duration of specialization, the average monthly number of births, the average daily number of patients examined, working conditions regarding the presence of a minor specialty, and their level of knowledge about umbilical cord clamping time during delivery, cord milking, skin-to-skin contact, application methods, and the protocols they use, if any, were questioned. The clamping time was questioned and categorized as follows: as soon as it was born, 0-30 sec, 30 sec-1 min, 1-2 min, and over 2 min, and until the pulsations in the cord ceased. Clamping in less than 30 seconds was considered as early clamping and over 30 seconds as delayed clamping [7]. Participants included in the study were divided into two groups according to their work status as in public or private hospitals, and the differences in practices of umbilical cord clamping, cord milking, and skin-to-skin contact were evaluated.

Statistical analysis

Statistical analysis was performed using the SPSS software (version 20, IBM Corp, Armonk, USA). The data are expressed as mean (SD) and in percentiles. The distribution of the variable data was determined using visual (histograms, probability plots) and analytical methods (Kolmogorov-Smirnov or Shapiro-Wilk’s test). The Mann-Whitney U test was used for non-parametric numerical data and Student’s t-test was used for parametric numerical data. Categorical data were compared through the use of the Chi-square test or Fisher’s exact test as applicable. For the multivariate analysis, the possible factors (duration of specialization, monthly number of births, monthly number of births, sex, age, working status in private sector) identified with univariate analyses were entered into the logistic regression analysis to determine further independent predictors of delayed clamping. A p-value less than 0.05 was considered significant.

## Results

This questionnaire was sent to 770 obstetricians. A total of 522 participants answered the questions in full, 165 refused to participate in the study, and 83 did not answer all questions. Of the 522 participants included in the study, the mean age was 38.7 ± 7.4, the average age of those working in public hospitals was 38.6 ± 8.1, and the average age of those working in private hospitals was 39 ± 4.8, and there was a significant difference between the groups (p=0.026). There were 300 (57.5%) female and 222 (42.5%) male participants and there was no significant difference between the groups in terms of gender (p=0.932). Physicians working in public hospitals had significantly higher monthly births (38.5 ± 53.2 and 21.7 ± 16.6) and the number of patients they examined daily (56.3 ± 26.6 and 22.6 ± 14.9) compared to those working in private hospitals (p<0.001). It was determined that the duration of the specialty of physicians working in public hospitals was significantly less than those working in private hospitals (p<0.001). Twelve (2.3%) of the participants had an oncology minor and 48 (9.2%) were perinatology minor specialists and all of these physicians were working in public hospitals (Table 1).

**Table 1:** Demographic features Data presented as mean ± SD and n (%).

		Total participants n=522	Public n=396	Private n=126	p
Age (years)		38.7 ± 7.4	38.6 ± 8.1	39 ± 4.8	0.026
Sex					0.932
	Female	300 (57.5)	228 (57.6)	72 (57.1)	
	Male	222 (42.5)	168 (42.4)	54 (42.9)	
Years in the profession		9.2 ± 7.7	9.2 ± 8.4	9.4 ± 4.7	0.014
The average monthly number of births		34.4 ± 47.6	38.5 ± 53.2	21.7 ± 16.6	<0.001
The number of daily examined patients		48.2 ± 28.3	56.3 ± 26.6	22.6 ± 14.9	<0.001
Minor specialty					0.005
	1.Yes (Oncology)	12 (2.3)	12 (3.0)	0 (0.0)	
	2.Yes (Perinatology)	48 (9.2)	48 (12.1)	0 (0.0)	
	3. No	462 (88.5)	336 (84.8)	126 (100.0)	

When the clamping styles applied by the participants were evaluated, it was determined that 288 (55.2%) clamp the umbilical cord late. It was found that the late clamping rates of physicians working in public hospitals were significantly lower than those working in private (p<0.001). It was determined that only 1.1% of the physicians used a common protocol in their institutions regarding the clamping procedure (Table 2). It was determined that 510 (99.7%) of the participants included in the study knew what cord milking was, 132 of them (25.3%) did not use the cord milking method at all, 210 (40.2%) used it sometimes, and 180 (34.5%) applied it always, and no significant difference was found between physicians working in the public and private sector (p=0.130).

**Table 2:** The relationship between umbilical cord clamping, milking, and skin-to-skin contact characteristics with the institution where specialists work Data presented as n (%); OR: odds ratio; Cl: confidence interval

		All participants	Public	Private	OR	%95Cl	p
Type of clamping					4.7	2.9-7.8	<0.001
	Early	234 (44.8)	210 (53.0)	24 (19.0)			
	Delayed	288 (55.2)	186 (47.0)	102 (81.0)			
Is there a protocol for cord clamping in your hospital?					-		0.330
	No	378 (72.4)	288 (72.7)	90 (71.4)			
	Yes, but each physician applies their own criteria	138 (26.4)	102 (25.8)	36 (28.6)			
	Yes, and everybody follows this protocol	6(1.1)	6 (1.5)	0 (0.0)			
Do you know what umbilical cord milking is?					0.8	0.7-0.9	0.052
	Yes	510 (97.7)	384 (97.0)	126 (100.0)			
	No	12 (2.3)	12 (3.0)	0 (0.0)			
Do you apply cord milking?					-		0.130
	Yes	180 (34.5)	144 (36.4)	36 (28.6)			
	Sometimes	210 (40.2)	150 (37.9)	60 (47.6)			
	No	132 (25.3)	102 (25.8)	30 (23.8)			
In which situations do you apply cord milking?					-		<0.001
	In term fetuses	54 (10.3)	42 (10.6)	12 (9.5)			
	In preterm fetuses	90 (17.2)	84 (21.2)	6 (4.8)			
	I apply it to avoid wasting time in cases where I want to do delayed clamping but cannot because of the necessity for neonatal resuscitation	108 (20.7)	66 (16.7)	42 (33.3)			
	All of the above	174 (33.3)	132 (33.3)	42 (33.3)			
	None	96 (18.4)	72 (18.2)	24 19.0)			
Do you keep the fetus inferior to the mother after birth?					-		0.538
	Sometimes	114 (21.8)	90 (22.7)	24 19.0)			
	Yes	204 (39.1)	150 (37.9)	54 42.9)			
	No	204 (39.1)	156 (39.4)	48 38.1)			
Do you apply 'skin-to-skin contact' between the fetus and the mother?					0.3	0.2-0.6	<0.001
	Yes	384 (73.6)	276 (69.7)	108 85.7)			
	No	138 (26.4)	120 (30.3)	18 14.3)			

Umbilical cord milking was performed by 174 physicians (33.3%) in all term, preterm, and fetuses requiring resuscitation, by 54 physicians (10.3%) only in term fetuses, by 90 physicians (17.2%) only in preterm fetuses, and by 108 (20.7%) physicians only in fetuses requiring resuscitation in order not to lose time. It was determined that physicians working in public hospitals applied this practice more (21.2% and 4.8%), especially in preterm fetuses, compared to those working in private. It was determined that 204 of the physicians (39.1%) always practiced keeping the baby below the maternal level immediately after birth, 114 (21.8%) sometimes did it, and 204 (39.1%) did not do it, and there was no statistically significant difference between the groups regarding this procedure (p=0.538) (Table 2).

It was observed that 384 (73.6%) of the physicians applied skin-to-skin contact between the fetus and the mother after birth, and 138 (26.4%) did not (Table 2).

Of the physicians included in the study, 48 (9.2%) stated that they clamped the cord immediately, 186 (35.6) did it within the first 30 seconds, 210 (40.2%) did it between 30s-1min, 48 (40.2%) did it between 1-2 minutes, and 12 (2.3%) stated that they clamped the cord when the pulsations ceased. Also, 132 of the physicians (25.3%) stated that they never practiced cord milking. One-hundred and eighty (34.5%) of the physicians who applied cord milking stated that they applied it once in every birth, 96 (18.4%) did it two times, and 114 (21.8%) did it three or more times (Table 3).

**Table 3:** Umbilical cord clamping time and milking frequency

		n	%
When do you often clamp the cord?	Immediately (as soon as the baby is born)	48	9.20%
	First 30 sec	186	35.60%
	30 sec-1 min	210	40.20%
	1-2 min	48	9.20%
	2 min or more	18	3.40%
	I wait until the cord pulsations are ceased	12	2.30%
How many times do you milk the umbilical cord?	0	132	25.30%
	1	180	34.50%
	2	96	18.40%
	3	102	19.50%
	4	12	2.30%

With multivariate regression analysis, it was determined that increased age, decreased years of expertise, decrease in the number of daily examined patients and monthly births, and working status in private hospitals increased the rate of delayed clamping (p<0.05). Multivariate logistic regression analysis was conducted with potential factors that may affect delayed clamping time. As a result, the decrease in the number of patients examined daily (OR=1.2, 95% CI=1.0-1.4) and working status in private hospitals (OR=3.6, 95% CI=2.0-6.3) were found to be the most important independent factors affecting delayed clamping (Table 4).

**Table 4:** Univariate and multivariate logistic regression model of potential factors (duration of specialization, monthly number of births, monthly number of births, sex, age, working status in private sector) affecting delayed clamping. OR: odds ratio; CI: confidence interval; B: coefficient for the constant; SE: standard error

	Multivariate					Univariate				
	B	SE	P	OR	95% CI	B	SE	P	OR	95% CI
Duration of specialization (years)	0.003	0.046	0.956	1.0	0.9-1.1	0.077	0.014	<0.001	1.1	1.1-1.2
Monthly number of births	0.003	0.002	0.185	1.0	0.9-1.0	-0.004	0.002	0.041	0.9	0.9-1.0
The number of daily examined patients	-0.010	0.005	0.039	1.2	1.0-1.4	-0.020	0.003	<0.001	0.9	0.9-0.9
Sex	0.150	0.201	0.454	1.1	0.7-1.7	-0.303	0.179	0.091	0.8	0.5-1.1
Age (years)	0.072	0.047	0.126	1.1	0.9-1.2	0.082	0.014	<0.001	1.1	1.1-1.1
Working status in private sector	1.278	0.290	<0.001	3.6	2.0-6.3	1.568	0.248	<0.001	4.8	2.9-7.8

## Discussion

In this study, it was determined that 55.2% of the participating obstetricians clamped the umbilical cord late. It was observed that the rates of delayed clamping increased with the age and professional experience of the participating specialists, and decreased with the increase in the number of monthly deliveries and the number of daily examined patients. Besides, this application was preferred more frequently by specialists working in private. It was observed that 25.3% of the doctors did not apply the cord milking at all and 17.2% applied it in preterm fetuses. It was seen that skin-to-skin contact was applied by 73.6%. It was determined that the working status of physicians in public or private institutions was the most important factor affecting these practices.

Active management of the third stage of labor was defined as the "cornerstone" of obstetrics and midwifery practices by the WHO in the second half of the 20th century [17]. Active management consists of three interrelated stages: (1) administration of a prophylactic uterotonic drug; (2) cutting the cord by clamping; (3) controlled traction of the umbilical cord. Birth practices have also changed in accordance with our changing and developing medical knowledge over the years. Although the timing of cord clamping often appears to be related to the preference of the obstetrician, WHO no longer recommends early cord clamping as a component of active management in its updated guideline, but recommends clamping 1-3 minutes after birth.

When the literature is reviewed, delayed clamping was detected as 90% in the study by Boere et al. conducted with 500 health professionals in the Netherlands, 67% in the study by Leslie et al. performed with 171 obstetricians in the USA, and 76% in the study conducted by Lundberg et al. with 50 obstetric departments in Norway [16, 18, 19]. In our country, the Ministry of Health recommends the active management of the third stage of labor but does not make any clear recommendations regarding the clamping time. In our study, it is seen that 55.2% of obstetricians prefer delayed clamping. We do not know the change in this rate over time, but when compared with the data of other countries, it is seen that obstetricians in our country prefer delayed clamping, although not at a high rate. In addition to delayed umbilical clamping, 39.1% of newborns are kept below the placental level (to increase placental transfusion with the effect of gravity) [20].

According to Turkish Population and Health Research (TPHR) 2018 data, 99% of births in our country are carried out in a health institution and mostly (59%) in public hospitals. Of all the births, 83% are performed by obstetricians and 16% by nurses or midwives [21]. In the study by Kurt et al [22], it was stated that midwives in our country frequently prefer early clamping, however, there is no study evaluating the practices of obstetricians. In our study, the factors that increase the rate of delayed clamping of the specialists were determined as the increasing age and professional experience, the low number of daily examined patients, and monthly births. On the contrary, in a study conducted in Spain, it was stated that increasing age and duration of expertise decrease the rate of delayed clamping [15]. This difference may be due to the variability in the socio-economic conditions of the countries, the prevalence in graduate education programs, and the change in the level of interest of physicians in childbirth practice. In the same study, it was observed that female healthcare professionals who delivered performed delayed clamping more frequently. In our study, no relationship was found between gender and clamping preference.

Another factor that is effective in clamping time is 'the hospital'. In the literature, it is seen that delayed clamping is preferred in large hospitals with 1000-2000 deliveries per year compared to hospitals with 1000 or fewer deliveries. However, it is stated that this rate decreases in hospitals with an annual birth rate of 4000 or more [15]. In our study, the decrease in delayed clamping rates with the increase in the number of deliveries can be interpreted as a similar result and we think that this situation may be related to patient density. As seen in our study, the number of daily patients that private working physicians take care of and their monthly births are lower than physicians working in public institutions, which also affects the birth practices. The fact that physicians working in private are older and they are more experienced in the field might be another reason. In the multivariate regression analysis performed in our study, working status in a private hospital was found to be the most important factor affecting delayed clamping rates. It is observed that specialists working in private tend to practice skin-to-skin contact more frequently. The intensity and some impossibilities in public hospitals direct families to give birth in private hospitals. According to the TPHR data, it is stated that private clinics are preferred for birth, especially in the western provinces with higher welfare [21].

Systematic reviews demonstrate the short and long-term benefits of delayed clamping. Increased hemoglobin values, the improvement of iron stores, and the reduction of necrotizing enterocolitis and intraventricular hemorrhage in preterms are among its main benefits. Besides, it is also stated that the timing of clamping does not have a great effect on blood loss during delivery [23]. Despite the thought that resuscitation may be delayed in preterm fetuses, many professionals who do not want to experience this situation apply the milking procedure by clamping the cord early. However, the potential benefits of cord milking are not yet clear. On the other hand, since gas exchange continues in the placenta after birth, it is thought that the additional blood volume provided by delayed clamping in premature babies may be more beneficial than the milking procedure. In studies on umbilical cord milking and cord clamping, it is observed that healthy analyses cannot be performed due to the small numbers in the compared subgroups. In only one study, it was noted that milking the cord four times had similar effects as doing the clamping 30 seconds later [24]. When the literature is examined, it is seen that a small number of obstetricians often milk the cord. It is seen that 17% of all healthcare professionals who deliver in Spain and only six out of 50 centers included in the study in Norway apply milking protocols [15, 19]. In our study, it was determined that 40.2% of the experts performed cord milking. It is seen that the cord is often milked once, and three or more milkings are applied at a rate of 24.1%. The tendency to milk the umbilical cord may be due to augmenting possible fetal benefit, as with late clamping.

The optimal time for clamping the umbilical cord is not yet clearly known [17, 18]. Although there is no evidence-based study, waiting until the pulsations cease in the cord was first defined by Darwin in 1801 and has come to this day after being accepted by many authorities [25]. In two recent studies, it has been reported that 54% and 69% of healthcare professionals wait until the pulses ceased in the cord [16, 26]. This rate was found to be 2.3% in our study. This different distribution in the literature can be attributed to the fact that waiting for cessation of arterial flow could not show the fetal benefits to be generated through the umbilical vein with evidence-based data. On the other hand, in the form of a new birth method called the "Lotus Birth", which was first applied in Australia in 2004, it waits until the placenta is separated from the mother, and the separation of the cord from the newborn by itself without clamping is monitored under appropriate conditions without intervention. Although the practitioners stated that the fetus is supported immunologically and spiritually, there is no evidence-based study on the subject [27].

This is the first study performed with obstetricians in our country on the practice of birth, cord clamping, milking, and skin-to-skin contact. In our country, obstetricians are the primary practitioners and are responsible for deliveries. For this reason, only the practices of obstetricians were taken into consideration and the residents and midwives were not included in the study in order not to create bias while the study was designed. This design makes the study powerful. However, it may constitute a limiting feature of the study that the participants may feel an unnoticed social pressure and did not answer the questions honestly. Another limitation is that the neonatal results of the applications cannot be evaluated.

## Conclusions

There are practical differences in terms of clamping and milking among healthcare professionals who deliver all over the world. It is seen that factors of health services and physicians are effective in the choice of clamping. In our study, increasing age, professional experience, and low patient density were found to be effective in increasing the rates of delayed clamping. However, the most important factor is whether the institution where physicians work is public or private. Although delayed clamping seems to be a recommended practice, due to the lack of an accepted protocol in our country, it is applied depending on the hospital conditions and the experts' preference. When all these factors are considered, the reasons for the practice of obstetrics that vary among other countries can be better understood. The widespread use of late clamping is related to the regulation of health policies and the development of applicable protocols. Providing more scientific evidence to persuade healthcare professionals of this issue can be a supporting force.
